# Cigarette smoking, *von Hippel–Lindau* gene mutations and sporadic renal cell carcinoma

**DOI:** 10.1038/sj.bjc.6603281

**Published:** 2006-08-01

**Authors:** B A C van Dijk, L J Schouten, E Oosterwijk, C A Hulsbergen-van de Kaa, L A L M Kiemeney, R A Goldbohm, J A Schalken, P A van den Brandt

**Affiliations:** 1 Department of Epidemiology, NUTRIM, Maastricht University, PO Box 616, Maastricht 6200 MD, The Netherlands; 2 Department of Urology, Radboud University Nijmegen Medical Centre, PO Box 9101, Nijmegen 6500 HB, The Netherlands; 3 Department of Pathology, Radboud University Nijmegen Medical Centre, PO Box 9101, Nijmegen 6500 HB, The Netherlands; 4 Department of Epidemiology and Biostatistics, Radboud University Nijmegen Medical Centre, PO Box 9101, Nijmegen 6500 HB, The Netherlands; 5 Department of Food and Chemical Risk Analysis, TNO Nutrition and Food Research, PO Box 360, Zeist 3700 AJ, The Netherlands

**Keywords:** renal cell carcinoma, cigarette smoking, *von Hippel–Lindau* gene mutations, The Netherlands, cohort study

## Abstract

We investigated whether smoking is associated with mutations in the *Von Hippel–Lindau (VHL)* gene in 337 cases of sporadic renal cell carcinoma (RCC) among 120 852 people followed for 11.3 years; the findings suggest that smoking causes RCC independently of *VHL* gene mutations.

Mutations in the *von Hippel–Lindau* (*VHL*) tumour suppressor gene are a common, early event in sporadic clear-cell renal cell carcinoma (RCC) (Gnarra *et al*, 1996; Richards *et al*, 1998). Tobacco smoke is implicated in the aetiology of RCC, with dose-dependent increased risks for former (rate ratio RR: 1.21; 95% CI: 1.07–1.36) and current (RR: 1.45; 95% CI: 1.26–1.66) smokers (Hunt *et al*, 2005). Cigarette smoke metabolites have been shown to cause specific DNA mutations (Harris, 1991; Shiao *et al*, 1998; Phillips, 2002), and are excreted in the urine (Hecht, 2002). Occupational exposure to trichloroethylene (Brauch *et al*, 1999, 2004) and consumption of citrus fruit and vegetables (confined to smokers) (Hemminki *et al*, 2002) have been linked to *VHL* mutations in RCC. We have investigated whether cigarette smoking is associated with sporadic RCC and with mutations in the *VHL* gene in clear-cell RCC in a prospective cohort study.

## MATERIALS AND METHODS

The Netherlands Cohort Study on diet and cancer (NLCS) included 120 852 men and women, aged 55–69 years, in 1986. The study was designed as a case–cohort study, using all cases and a random sample of 5000 persons from the cohort (subcohort) (Van den Brandt *et al*, 1990a). All participants who reported prevalent cancer (excluding skin cancer) at baseline were excluded from analyses (leaving 4774 subcohort members). The subcohort was followed to estimate the accumulated person-years in the entire cohort (Volovics and van den Brandt, 1997). Follow-up for incident cancer was established by computerised record linkage with the Netherlands Cancer Registry (NCR) and PALGA, a national database of pathology reports (Van den Brandt *et al*, 1990b), and was estimated to be more than 96% complete (Goldbohm *et al*, 1994b). From 1986 to 1997 (11.3 years follow-up), 355 kidney cancer cases (ICD-O-3: C64.9) were identified. Urothelial cell carcinomas were excluded and only histologically confirmed epithelial cancers were included (ICD-O: M8010–8119, 8140–8570), leaving 337 cases.

The collection, classification, DNA isolation and mutation analyses have been described previously (van Houwelingen *et al*, 2005). After revision and *VHL* mutation analyses, data were available for 235 cases.

At baseline, all cohort members completed a mailed, self-administered questionnaire on dietary habits (food-frequency questionnaire), lifestyle, smoking, personal and family history of cancer and demographic data (Goldbohm *et al*, 1994a). Questions on cigarette smoking in the questionnaire addressed smoking status (never, ex- or current smoker), age at first and last exposure, frequency and duration. Information on smoking status was complete for 336 cases and 4762 subcohort members.

Differences between cases with and without collected tumour material, between men and women and between cases with and without a mutation in the *VHL* gene were assessed by Student's *t*-tests and *χ*^2^ tests. RRs and corresponding 95% confidence intervals (CI) were estimated using Cox proportional hazard models processed with STATA (STATA statistical software, Release 7, STATA Corporation, College Station, TX, USA, 2001), after testing the proportional hazards assumption using scaled Schoenfeld residuals (Schoenfeld, 1982). Standard errors were estimated using the robust Huber–White sandwich estimator to account for additional variance introduced by sampling from the cohort (Lin and Wei, 1989). To obtain *P*-values for dose–response trends, ordinal exposure variables were fitted as continuous terms. Two-sided *P*-values are reported throughout this paper.

RRs were calculated for never, ex- and current smokers for the total group and for men and women separately. The following case groups were defined: total RCC (all cases of RCC; *N*=337); clear-cell RCC (all cases of RCC classified as clear-cell by one experienced pathologist (CAHK); *N*=187); *VHL*-mutated clear-cell RCC (clear-cell RCC with a mutation in the *VHL* gene; *N*=114) and *VHL* wild-type clear-cell RCC (clear-cell RCC without a mutation in the *VHL* gene; *N*=73).

Because G:C → T:A transversions and G:C → A:T transitions have been linked to specific tobacco smoke constituents (Harris, 1991; Shiao *et al*, 1998), the association of smoking and these mutations was investigated.

Confounders entered in the analyses were age, sex (if appropriate) and body mass index. Cigar and pipe smoking (never, ex- and current smoker) were additionally included in the model to account for other sources of tobacco smoke. To assess dose–response relations, the number of cigarettes smoked per day was categorised into <10, 10–20, 20–30 and 30 or more cigarettes per day (additionally adjusted for smoking years), and the number of smoking years was categorised into <20, 20–40 and 40 or more smoking years (additionally adjusted for cigarettes smoked per day). Age at first exposure (<15, 15–17, 17–20 and 20 or older) and years of cessation (<5, 5–20 or more than 20 years) were also investigated, with additional adjustment for cigarettes smoked per day.

## RESULTS

Table 1 shows baseline characteristics. There were no statistically significant differences between cases with (*N*=235) or without (*N*=102) tumour tissue. The mean age was somewhat higher for cases than for subcohort members (Table 1). There were almost no male never smokers, whereas female smokers were scarce.

Table 2 shows multivariable adjusted analyses for smoking for the earlier described case groups. Statistically significantly increased RRs were observed for men only (Table 2). The RRs were higher for *VHL* wild-type clear-cell tumours than for mutated clear-cell tumours. This was observed both in men and women.

Dose–response effects were indicated (increasing risk with increasing smoking frequency and a lower risk of RCC after cessation without a clear trend). There were no noteworthy differences between mutated and wild-type clear-cell RCC. This was investigated in ever-smoking men only, as the number of women was too low for meaningful analyses (van Dijk *et al*, 2006).

The distribution of smokers for the different type of mutations, the mutational spectra, specific mutations (i.e. G:C → T:A transversions and G:C → A:T transitions) and location of mutations was not different for never, ex- and current smokers (van Dijk *et al*, 2006).

## DISCUSSION

Our results indicate that smoking was associated with increased risk of RCC, but that the number of mutations in the *VHL* gene was not increased by smoking.

The percentage of smokers in this cohort appears to be slightly lower compared to the percentage in the population, which may either be the result of a selective response by smoking status to the baseline questionnaire or of under-reporting of smoking habits because of social desirability. The response rate to the questionnaire at baseline equalled 35.5% (Van den Brandt *et al*, 1990a), with a slight shift towards nonsmoking compared to the population (Goldbohm *et al*, 1991). This selective response would not lead to altered RRs, whereas under-reporting would result in an underestimation of the RRs.

Latency of RCC is unknown, but a latency of decades seems plausible. Most of the ever smokers in the subcohort had smoked for 20 years or more (86.0% of men and 75.5% of women), whereas 42.2% of men and 23.7% of women had smoked for 40 years or more. Also, 77.0% of men and 36.7% of women started smoking before the age of 19. These percentages are sufficiently high to assume that smoking could have caused cancer within the time frame measured by this study.

We hypothesised smoking to be associated with *VHL* mutations. Contrary to what we expected, RRs were somewhat higher for *VHL* wild-type tumours than for *VHL*-mutated tumours. No association of smoking and specific types of mutations was found. The observation that exposure to BPDE (a smoke constituent) induces G:C → T:A transversions has predominantly been reported for the p53 tumour suppressor gene in lung cancer (Hussain *et al*, 2001) and may not apply to all genes and cancer sites, for example, not one p53 G:C → T:A transversion was observed in smokers in a study on bladder cancer (Spruck *et al*, 1993). Exposure to *N*-nitrosamino compounds was associated with G : C → A : T transitions in the RAS gene in tumours of rodents (Harris, 1991) and also with *VHL* mutations in rats (Shiao *et al*, 1998). We found no evidence to suggest that this plays a role in humans.

We are among the first to investigate the association of risk factors with *VHL* mutations. A direct association between a risk factor and mutations may give additional information on the pathway(s) that lead to tumour growth. Previously, a positive association of occupational exposure to trichloroethylene, an industrial solvent, to *VHL* mutations and a hot spot for mutations was observed in a case–control study (Brauch *et al*, 1999, 2004). However, long-term exposure to an extremely high dose of the probable carcinogen trichloroethylene was investigated. Decreased risks were observed for vegetable consumption (for smokers) and citrus consumption (for smokers and nonsmokers together) and mutations in a case-only study (Hemminki *et al*, 2002). In this report, the OR for risk of *VHL* mutations compared to wild-type *VHL* as a result of smoking equalled 0.95 (95% CI: 0.41–2.21) (Hemminki *et al*, 2002). This supports our observation that smoking may not be associated with *VHL* mutations.

Smoking was associated with RCC risk for men, but smoking was not associated with *VHL* mutations, irrespective of sex, implying that smoking may cause or promote RCC independent from *VHL* mutations.

## Figures and Tables

**Table 1:** Description of possible confounding variables and smoking variables for men and women, Netherlands Cohort Study on diet and cancer, 1986–1997

	**Men**			**Women**		
**Variable**	**Subcohort,** ***N*=2331**	**RCC cases,** ***N*=217**	**RCC, tumour tissue, collected** ***N*=148**	**Subcohort,** ***N*=2431**	**RCC cases,** ***N*=119**	**RCC, tumour tissue, collected** ***N*=87**
Age, mean (s.d.)	61.4 (4.2)	62.0 (3.8)	62.2 (3.8)	61.5 (4.3)	61.8 (4.0)	61.6 (4.0)
						
*Family history of RCC*						
Yes, *N* (%)	14 (0.6)	3 (1.4)	2 (1.4)	33 (1.4)	1 (0.8)	1 (1.2)
						
*History of hypertension*						
Yes, *N* (%)	534 (22.9)	56 (25.8)	37 (25.0)	700 (28.8)	41 (34.5)	31 (35.6)
						
BMI, mean (s.d.)	25.0 (2.6)	25.3 (2.7)	25.4 (2.6)	25.1 (3.6)	25.8 (3.4)	25.7 (3.2)
	(*N*=2247)	(*N*=210)	(*N*=141)	(*N*=2341)	(*N*=113)	(*N*=82)
*Cigarette smoking*						
Never, *N* (%)	300 (12.9)	18 (8.3)	12 (8.1)	1431 (58.9)	66 (55.5)	49 (56.3)
Ex, *N* (%)	1175 (50.4)	108 (49.8)	79 (53.4)	491 (20.2)	21 (17.6)	15 (17.2)
Current, *N* (%)	856 (36.7)	91 (41.9)	57 (38.5)	509 (20.9)	32 (26.9)	23 (26.4)
						
*Never smoker*						
Yes *N* (%)	217 (9.3)	11 (5.1)	6 (4.1)	1431 (58.9)	64 (53.8)	48 (55.2)
						
*Cigarette only smoker*						
Yes, *N* (%)	1413 (60.6)	136 (62.7)	86 (58.1)	995 (40.9)	53 (44.5)	38 (43.7)
						
*Cigar and/or pipe smoker*						
Yes, *N* (%)	83 (3.6)	7 (3.2)	6 (4.1)	0	2 (1.7)	1 (1.2)
						
*Cigarette and other type of tobacco smoker*						
Yes, *N* (%)	618 (26.5)	63 (29.0)	50 (33.8)	5 (0.2)	0	0

Abbreviations: BMI, body mass index; RCC, renal cell carcinoma; s.d., standard deviation.

**Table 2:** Rate ratios for ex- and current smokers compared to never smokers for all tumours (total), clear-cell tumours, clear-cell tumours with a *VHL* gene mutation and *VHL* wild-type clear-cell cases, Netherlands Cohort Study on diet and cancer (1986–1997)

	**Never smokers**			**Ex-smokers**			**Current smokers**			
	**Cases (*N*)**	**Person-years subcohort**	**RR^a^**	**Cases (*N*)**	**Person-years subcohort**	**RR (95% CI)**	**Cases (*N*)**	**Person-years subcohort**	**RR (95% CI)**	***P*-value for interaction^b^**
*Men and women* c										
Total	81	17 906	1	125	16 759	1.17 (0.85–1.61)	117	13 377	1.60 (1.17–2.20)	0.43
Clear-cell	48	17 906	1	73	16 759	1.34 (0.91–1.99)	57	13 377	1.49 (0.98–2.26)	0.20
Clear-cell wild-type	18	17 906	1	27	16 759	1.58 (0.84–2.94)	26	13 377	2.06 (1.07–3.94)	0.55
Clear-cell mutated	30	17 906	1	46	16 759	1.20 (0.73–1.96)	31	13 377	1.17 (0.69–1.99)	0.24
										
*Men* d										
Total	17	3086	1	105	11 643	1.52 (0.89–2.59)	88	8176	2.07 (1.20–3.56)	
Clear-cell	6	3086	1	60	11 643	2.43 (1.07–5.56)	39	8176	2.54 (1.05–6.17)	
Clear-cell wild-type	2	3086	1	21	11 643	2.68 (0.66–10.86)	15	8176	2.95 (0.65–13.28)	
Clear-cell mutated	4	3086	1	39	11 643	2.33 (0.84–6.44)	24	8176	2.34 (0.79–6.94)	
										
*Women* e										
Total	64	14 820	1	20	5116	0.95 (0.57–1.59)	29	5201	1.37 (0.87–2.16)	
Clear-cell	42	14 820	1	13	5116	0.94 (0.50–1.76)	18	5201	1.29 (0.73–2.28)	
Clear-cell wild-type	16	14 820	1	6	5116	1.12 (0.44–2.86)	11	5201	2.04 (0.94–4.45)	
Clear-cell mutated	26	14 820	1	7	5116	0.82 (0.35–1.92)	7	5201	0.82 (0.35–1.93)	

Abbreviations: RCC, renal cell carcinoma; RR, rate ratio; s.d., standard deviation; VHL, Von Hippel–Lindau.

a Reference group.

b Test for interaction of sex and smoking.

c Cigarette, cigar and pipe smoking, multivariable adjusted for age, sex and BMI.

d Cigarette, cigar and pipe smoking, multivariable adjusted for age and BMI.

e Cigarette smoking, multivariable adjusted for age and BMI.
